# *Megarthrus* of China. Part 4. The *M.hemipterus* complex (Coleoptera, Staphylinidae, Proteininae), with description of a new species from Yunnan Province

**DOI:** 10.3897/zookeys.1056.66553

**Published:** 2021-08-17

**Authors:** Zhiping Liu, Giulio Cuccodoro

**Affiliations:** 1 Institute of Entomology, College of Plant Protection, Southwest University, Chongqing 400715, China Southwest University Chongqing China; 2 Muséum d’histoire naturelle, Case postale 6434 CH-1211, Genève 6, Switzerland Muséum d’histoire naturelle Geneva Switzerland

**Keywords:** Endemism, *Megarthrusdepressus* supergroup, morphology, taxonomy

## Abstract

The members of the *Megarthrushemipterus* species complex occurring in China, i.e., *M.dentipes* Bernhauer, *M.flavolimbatus* Cameron and *M.hemipterus* (Illiger), are diagnosed, and a new species attributed to this informal group, *M.panda***sp. nov.**, is described from Yunnan Province. All species are diagnosed and illustrated, and their distribution in mainland China is mapped. The limit of the *M.hemipterus* species complex is refined morphologically.

## Introduction

This paper is the fourth of our series aiming at the taxonomic treatment of the fauna of *Megarthrus* Curtis, 1929 of mainland China, which consists at present of twelve species (Liu and Cuccodoro 2020a, b; Zhang et al. 2021).

Here, we deal with the members of the *M.hemipterus* species complex, i.e., *M.dentipes* Bernhauer, 1938, *M.flavolimbatus* Cameron, 1924, and *M.hemipterus* (Illiger, 1794), which form a subset of the speciose and predominantly Palaearctic, Nearctic and African *M.depressus* supergroup (Cuccodoro and Löbl 1997; Cuccodoro 2003, 2011). We also describe a new species from Yunnan Province in China, which we place in the *M.hemipterus* complex along with a refinement of the delimitation of the latter. These four species are diagnosed and illustrated, and their distribution in mainland China is mapped. A key to the Chinese species of the *M.depressus* supergroup will be provided only after the more than ten still undescribed Chinese species of this lineage will be treated.

## Materials and methods

The material treated in this study is deposited in the following collections:

**cAss** Volker Assing private collection (Hannover, Germany).

**cPüt** Andreas Pütz private collection (Eisenhüttenstadt, Germany).

**cSch** Michael Schülke collection (Museum für Naturkunde Berlin, Germany).

**FMNH**Field Museum of Natural History, Chicago.

**MHNG**Muséum d’histoire naturelle, Geneva, Switzerland.

**NHMB**Naturhistorisches Museum, Basel, Switzerland.

**NMPC**National Museum of Natural History, Prague, Czech Republic.

**SWUC** Institute of Entomology, College of Plant Protection, Southwest University, Chongqing, China.

For detailed examination, specimens were relaxed in water prior dissection. Dissected body parts were dehydrated in isopropanol (genitalia after clearing in aqueous 0.1 N solution of potassium hydroxide) and mounted in Canada Balsam on acetate slides. Drawings were made by using a drawing tube mounted on a compound microscope. The habitus images were taken using a Leica DFC425 camera in conjunction with a Leica M205–C compound microscope. Images of morphological structures were made using a Canon G9 camera mounted on a Zeiss Axioscope 50 microscope. Zerene Stacker (version 1.04) was used for image stacking. All images were modified and grouped in Adobe Photoshop CS5 Extended (version 12.0). The distribution map was captured from Google Earth.

Abdominal sternites and tergites are counted from the first morphological segment and quoted in Roman numbers (i.e., last visible tergite = tergite VIII).

## Taxonomy

The species treated below all share the following features typical of the *M.depressus* supergroup (Cuccodoro 2011): antennal pubescence markedly denser on antennomeres 5–11 than on antennomeres 1–4; metaventral setae shorter than proventral setae; abdominal sternites each bearing two posteromedial macrosetae; maxillary palpus with palpomere 3 about 1.5 times as long as palpomere 2; occipital ridge indistinct; antennomere 11 ovoid; pronotal hypomerom lacking marked ridge; male protarsomere 1 with adventral patch of modified tenent setae.

In addition they also share following characters: dorsal pubescence fairly uniform, slightly sparser on elytra than on pronotum and abdomen; pubescence on frons converging, with medial setae directed posteriad; anterior margin of frons slightly carinate, evenly convex in dorsal view; eyes hemispherical, with highest point above level of vertex; temples forming small sharp angle right behind eyes, and posteriorly abruptly narrowed, almost flat and fairly smooth; scape piriform, moderately compressed, about twice longer than wide; pronotum moderately deplanate, moderately convex in frontal view, and weakly convex in lateral view; lateral pronotal margins slightly raised on entire length, in dorsal view gently subangled at middle and strongly subangled subbasally, forming obsolete laterobasal inscision; medial groove well-marked on entire length; elytron slightly expanded posterior to humeral angle, shallowly depressed along lateral margin; lateral margin slightly carinate and denticulate, fairly straight or gently arcuate on posterior three-quarters in both dorsal and lateral views; male mesofemur longer than metafemur; male metatibia about as long as mesotibia; male abdominal sternite VIII similar to that in Fig. 32, lacking hyaline medial disc; hemitergites IX similar to that in Fig. 39, with lateral lobe moderately developed; aedeagus symmetrical, with dorsal valve elongate extended anteriorly beyond level of apex of parameres; base of parameres projecting posteriorly above ventral wall, forming marked cavity; female abdominal tergite VIII in lateral view with lateral margin fairly straight to apex acutely angled, not forming medioapical projection; female abdominal sternite VIII similar to that in Fig. 40, with posterior margin broadly arcuate; valvifers not meeting each other dorsally; gonocoxal plate compressed dorsoventrally, almost twice broader in dorsal view than in lateral view, lacking medioventral and mediodorsal ridges.

In order to keep the text more concise these characters will not be repeated in their respective descriptions.

### Megarthrus dentipes

Taxon classificationAnimaliaColeopteraStaphylinidae

Bernhauer, 1938

428115CE-D7F5-538B-896B-B37869F860AA

**Figures 1–3.**
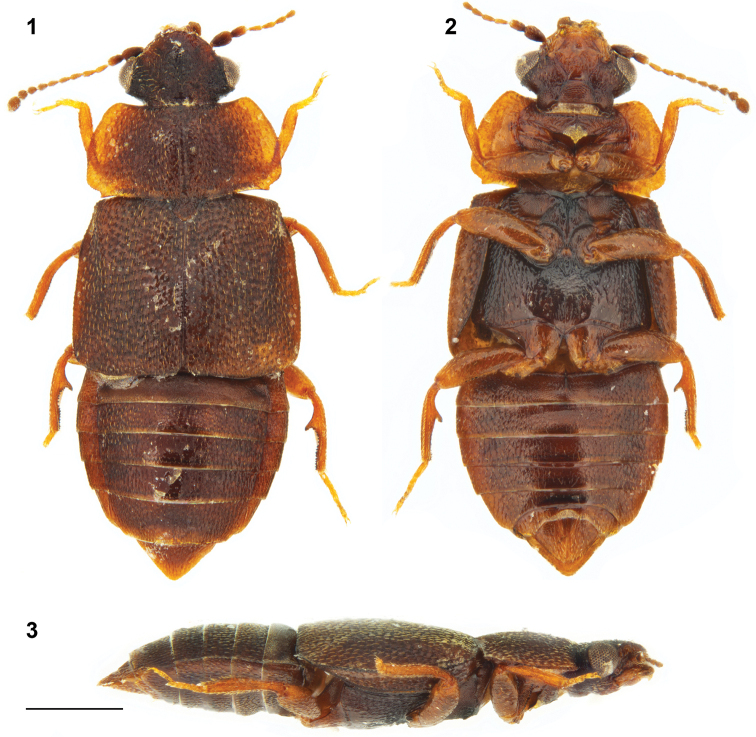
*Megarthrusdentipes* Bernhauer: habitus, male: dorsal **1** ventral **2** and lateral **3** views. Scale bar: 0.5 mm.

Figs 1–3 , 10 , 13 , 16 , 41

Megarthrus dentipes Bernhauer, 1938: 17; Cuccodoro and Löbl 1997: 1364 (detailed redescription).

#### Diagnosis.

For detailed morphology see Cuccodoro and Löbl (1997). Combined length of head, pronotum, and elytra 1.6–2.0 mm; maximal pronotal width = 0.8–1.1 mm. Body (Figs 1–3) predominantly chestnut brown, with pronotum slightly paler along lateral edges, frons slightly paler than vertex, and legs slightly paler than elytra. Anterior frontal margin slightly carinate, evenly convex in dorsal view.

***Male*.** Protibia slightly arcuate and evenly expanding from base to apex; adventral side broadly depressed transversely. Mesotrochanter with about a dozen of peg-like setae arranged in two rows. Mesofemur slightly arcuate and swollen. Mesotibia slightly arcuate, bearing peg-like setae arranged in rows. Metatrochanter and metafemur slightly swollen; posterior margin of metatrochanter evenly arcuate; inner margin of metafemur fairly straight in ventral view, forming sharp ridge on entire length. Metatibia swollen, at middle forming conspicuous tooth-like process projecting above flattened apical portion of metatibia; peg-like setae grouped as dense field on apical third, and extending in fairly continuous row to apex of distal side of tooth-like process; proximal side of tooth-like process convex, with at most 2 peg-like setae. Aedeagus (Figs 10, 13) with ventral wall slightly narrowed at base and evenly narrowed to blunt right-angled apex in ventral view, and with ventral outline slightly sinuate to apex slightly recurved ventrally in lateral view.

***Female*.** Gonocoxal plate with lateral portions of dorsobasal margin concave to middle portion subangled, markedly projecting anterad. Dorsal part of genitalia (Fig. 16) with evenly thick semi-circular sclerite.

#### Comparisons and diagnostic notes.

*Megarthrusdentipes* resembles in most characters of *M.flavolimbatus* and *M.hemipterus*. These species can be distinguished by the shape of the anterior frontal margin (i.e., evenly arcuate in *M.dentipes* and *M.hemipterus*, while more convex at middle than laterally in *M.flavolimbatus*) and the coloration (i.e., head fairly concolor with pronotum, and legs paler than elytra in *M.dentipes* and *M.flavolimbatus*, instead of markedly darker than the pronotum, and legs concolorous with the elytra in *M.hemipterus*). The males also differ by the shape and vestiture of the metatibial tooth-like process (i.e, its proximal side is flattened in *M.hemipterus* instead of convex in *M.dentipes* and *M.flavolimbatus*, and bearing peg-like setae on its distal side in a fairly continuous row to the apex in *M.dentipes*, while discontinuoulsy in *M.flavolimbatus* and *M.hemipterus*). The shape of the apical portion of the aedeagus ventral wall is diagnostic (i.e., subangulate in *M.dentipes* (Fig. 10), angulate in *M.flavolimbatus* (Fig. 11), and mucronate in *M.hemipterus* (Fig. 12)), as well as that of the medial portion of the dorsobasal margin of the gonocoxal plate (i.e., truncate and projecting anterad in *M.flavolimbatus*, subangled and projecting anterad in *M.dentipes*, and mucronate in *M.hemipterus*).

#### Material examined.

(78 specimens): **China: Fujian Prov.**: Guadun, “Kuatun, Fukien, Tschung Sen [sic], 5.iv.1946, leg J. Klapperich”, 1 ♂ in NHMW; 10 km E Yong’an, 25°58'N, 117°27'E, 31.v.2008, 700 m, leg. J. Tuma, 2 ♀ in NHMW; Fenshui Guan, 27.9N, 117.85E, 7.v.2005, 1700 m, leg. J. Tuma, 10 ♂ and 13 ♀ in NHMW, MHNG & ZMUC; 2km SE Xinqian, 27.05N, 117.1E, 10.v.2005, 1700 m, leg. J. Tuma, 4 ♂ and 6 ♀ in NHMW & MHNG; Ziyungdongshan, NW slopes, 25°46'N, 117°20'E, 25.iv.2006, 900–1000 m, leg. J. Tuma, 1 ♂ and 3 ♀ in NHMW; **Guangdong Prov.**: Nanling National Nature Reserve, Dadongshan, 24°54.7'N, 112°43.1'E, 770 m, 20–21.iv. 2013, leg. J. Hájek 6 J. Růžička, 1 ♂ in NHMP; **Hubei Prov.**: Mulu Shan, Jiugongshan forest park, 29.4N 114.6E, up to 1000 m, 3.v–18.vi.2002, leg. J. Tuma, 2 ♂ and 4 ♀ in NHMW; **Hunan Prov.**: Shunhuangshan forest park, 26°22–23'N, 111°00–01'E, 20.vi.2013, 1300–1600 m, leg. Jatua, 5 ♂ and 4 ♀ in NHMW & MHNG; **Jiangxi Prov.**: Jinggangshan Mts, Xiangzhou (forested valley S of the village), 26°35.5'N, 114°16.0'E, 374 m, (steam valley), 26.iv.2011, leg. Fikáček and Hájek, Jia and Song, [MF08] cut and decaying tops of bamboo trunks at the side of a trail in the secondary forest and among the fields, 1 ♂ and 2 ♀ in NHMP; Jinggangshan Mts, Songmuping, 26°34.7'N, 114°04.3'E, 1280 m, (stream valley), 27.iv.2011, leg. Fikáček, Hájek, Jia and Song [MF10] cut and decaying tops of bamboo trunks in a sparse bamboo bush, 7 ♂ and 6 ♀ in NHMP, MHNG & ZMUC; Jinggangshan Mts, Huyagta, 26°29.9'N, 114°07.3'E, 1490 m, 28.iv.2011, leg. Fikáček, Hájek, Kubeček, Jia, Song and Zhao, [MF12] cut and decaying tops of bamboo trunks in a sparse secondary bamboo forest, 2 ♂ and 2 ♀ in NHMP; **Zhejiang Prov.**: Baima Shan, 28°37'N, 119°09'E, 7–17.vi.2008, 1270–1520 m, leg. J. Tuma, 1 ♂ and 1 ♀ in NHMW.

#### Distribution and natural history.

Till now *M.dentipes* was known only from the Chinese Provinces of Jiangsu and Zhejiang (Cuccodoro and Löbl 1997: 1364). The new materials examined indicate that the species occurs in Zhejiang Province also at Baima Shan, as well as in several other localities of Hubei, Fujian, Jiangxi, and Guangdong Provinces (Fig. 41), where it was collected at elevations ranging from 374 to 1700 m a. s. l., mainly in rotten bamboo trunks and decaying cut tops of bamboo trunks.

### Megarthrus flavolimbatus

Taxon classificationAnimaliaColeopteraStaphylinidae

Cameron, 1924

FFA430E3-8FB9-531B-B9F6-5FB414D9C05B

**Figures 4–6.**
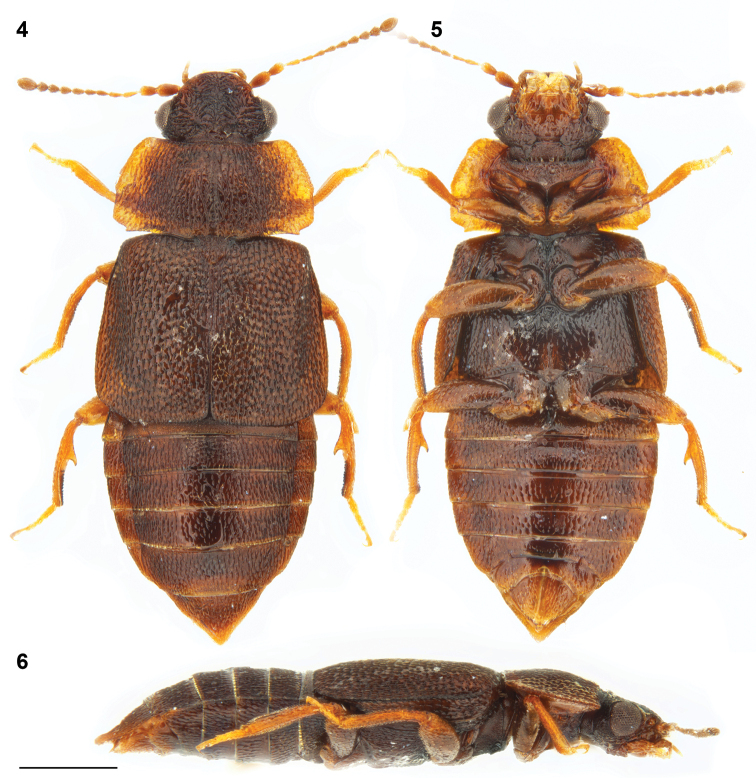
*Megarthrusflavolimbatus* Cameron: habitus, male: dorsal **4** ventral **5** and lateral **6** views. Scale bar: 0.5 mm.

Figs 4–6 , 11 , 14 , 17 , 41

Megarthrus flavolimbatus Cameron, 1924: 164; Cuccodoro 2003: 377 (detailed redescription, the first record for China); Cuccodoro 2011: 38 (photograph of dorsal habitus, Taiwanese records in China).

#### Diagnosis.

For detailed morphology see Cuccodoro (2003). Combined length of head, pronotum, and elytra 1.6–1.9 mm; maximal pronotal width = 0.9–1.1 mm. Body (Figs 4–6) predominantly chestnut brown, with pronotum slightly paler along lateral edges, frons slightly paler than vertex, and legs slightly paler than elytra. Anterior frontal margin slightly carinate, more convex at middle than laterally in dorsal view.

***Male*.** Protibia fairly straight and evenly expanding from base to apex; adventral side shallowly depressed transversely. Mesotrochanter with about a dozen peg-like setae arranged in two rows. Mesofemur fairly straight and slightly swollen. Mesotibia subangulate, bearing peg-like setae arranged in one row. Metatrochanter and metafemur slightly swollen; posterior margin of metatrochanter evenly arcuate; inner margin of metafemur fairly straight in ventral view, forming sharp ridge on entire length. Metatibia swollen, at middle forming conspicuous tooth-like process projecting above flattened apical portion of metatibia; metatibial peg-like setae grouped as a dense field on apical third, discontinuously with 1 peg-like seta to at most 7 peg-like setae on distal side of tooth-like process; proximal side of tooth-like process convex, with at most 1 peg-like seta. Aedeagus (Figs 11, 14) with ventral wall slightly narrowed at base and evenly narrowed to acutely angled apex in ventral view, and with ventral outline fairly straight to slightly recurved apex in lateral view.

***Female*.** Gonocoxal plate with lateral portions of dorsobasal margin with oblique to middle portion truncate, markedly projecting anterad. Dorsal part of genitalia (Fig. 17) with evenly thick semi-circular sclerite.

#### Comparisons and diagnostic notes.

See above under *M.dentipes*.

#### Material examined.

(92 specimens): **China: Fujian Prov.**: N of Wutongyang, 26°03'N, 117°38'E, 12.v.2010, 1000–1700 m, leg. J. Tuma, 25 ♂ and 9 ♀ in MHNG, NHMW & ZMUC; **Hunan Prov.**: Shunhuangshan Forest Park, 26°24'N, 111°01'E, 22.v.2013, 700–1200 m, leg. Jatua, 6 ♂ and 7 ♀ in MHNG & NHMW; **Shaanxi Prov.**: Qin Ling Shan, river valley 40 km S Xian, highway km 50, 33.55N, 108.49E, 1200 m, 31.viii.1995, leg. D. W. Wrase, river bank, 1 ♂ in cSch; **Sichuan Prov.**: Qingcheng Shan, NW Chengdu, 650–700 m, 30°53'57"N, 103°32'23"E, 3–4.vi.1997, leg. M. Schülke (17), 1 ♂ and 2 ♀ in cSch; Qingcheng Shan, 65 km NW Chengdu, 8 km W Taiping, 35.53N, 103.33E, 800–1000m, 18.v./3–4.vi.1997, leg. A. Pütz, 2 ♂ and 2 ♀ in cPüt & MHNG; Emei Shan, 29°33'04"N, 103°21'19"E, 25.vi.2011, 1729 m, leg. V. Grebennikov (sift 05) 2 ♂ and 1 ♀ in MHNG; Emei Shan, 29°36'06"N, 103°20'06"E, 27.vi–.5.vii.2009, 1800–2400 m, leg. V. Grebennikov (siftings 11–17) 2 ♂ and 1 ♀ in MHNG; Ya’an Pref., Shimian Co. Xiaoxiang Ling, 11 km S Shimian, side-valley above Nanya Cun near Caluo, 1250 m, 7. VII.1999, leg. A. Pütz, 1 ♀ in cPüt; **Yunnan Prov.**: Baoshan Pref., Gaoligong Shan, E pass, 36 km SE Tengchong, 24°49'32"N, 98°46'06"E, 2200 m, 31.v.2007, leg. A. Pütz, [CH07–13] deciduous forest, litter, wood, fungi sifted, 2 ♂ in cPüt; Baoshan Pref., Gaoligong Shan, 36 km SE Tengchong, 24°51'22"N, 98°45'36"E, 2100–2200 m, 31.v.2007, leg. A. Pütz [CH07–14], deciduous forest, litter, wood, fungi, sifted, 1 ♂ in cPüt; Baoshan Pref., mountain range 14 km E Tengchong, 25°00'28"N, 98°38'07"E, 1850 m, 1.vi.2007, leg. A. Pütz [CH07–16] second, mixed forest, litter sifted, 1 ♂ in cPüt; Baoshan Pref., mountain range 25 km S Tengchong, 24°48'28"N, 98°32'03"E, 1900 m, 2.vi.2007, leg. A. Pütz [CH07–17], dev. primary deciduous forest, litter, fungi, sifted, 1 ♀ in cPüt; Ruili, 4.ii.1993, G. de Rougemont, 2 ♂ and 2 ♀ in MHNG & SCNU; Xishuangbanna, 22.i.1993, G. de Rougemont, 2 ♂ and 3 ♀ in MHNG; Kunming, 9.x.1985, G. de Rougemont, 1 ♀ in MHNG; Kunming, x.1986, G. de Rougemont, 1 ♂ in MHNG; Baoshan Pref., Gaoligong Shan, E pass, 36 km SE Tengchong, 2200 m, 24°49'32"N, 98°46'06"E, 4.vi.2007, leg. M. Schülke [CH07–13], deciduous forest, litter, wood, fungi, sifted, 4 ♂ and 1 ♀ in cSch; Xishan Mts, 24.57N 102.38E, 2300 m, 27.vi.1993, leg. V. Kubáñ, 1 ♂ in NHMB; NE Kunming, 25°08'40"N, 102°53'48"E, 2290 m, 11. VIII. 2014, leg. V. Assing [5] mixed forest, sifted, 1 ♂ in cAss; NE Kunming, Xiaobailong Forest Park, 24°55'43"N, 103°05'27"E, 2110 m, 10.viii.2014, leg. M. Schülke [CH14–03], secondary pine forest margin, litter, sifted, 3 ♀ in cSch; Dali Bai Aut. Pref., Wuliang Shan, 9km SW Weishan, 25°10'15.5"N, 100°14'21.8"E, 2480 m, 14.ix.2009, leg. M. Schülke [CH09–51], scrub with (oak, alder, pine) litter and mushrooms, sifted, 2 ♀ in cSch; Baoshan Pref. Mountain range 25 km S Tengchong, 24°48'28"N, 98°32'03"E, 1900 m, 2.vi.2007, leg. M. Schülke, [CH07–17], dev. primary deciduous forest, litter, fungi, sifted, 1 ♀ in cSch; Gaoligong Shan, Pass SW Baoshan, 4–8.vi.2006, leg. Jeniš, 1 ♀ in cAss.

#### Distribution and natural history.

*Megarthrusflavolimbatus* is the most widespread member of the genus in the Oriental Realm, with records ranging from North India (Himachal Pradesh and West Bengal) to Yunnan Province and Taiwan in China (Cuccodoro 2003, 2011); here, we report it for the first time also from Fujian, Hunan, Shaanxi and Sichuan Provinces in China (Fig. 41). It was found at elevations ranging from 250 to 3375 m a. s. l. in Taiwan (Cuccodoro 2011), and its altitudinal range appears quite wide also in mainland China (i.e., from 1000 to 1700 m a. s. l. in Fujian, from 7000 to 1200 m a. s. l. in Hunan, from 1850 to 2480 m a. s. l. in Yunnan, from 650 to 2400 m a. s. l. in Sichuan, and at 1200 m a. s. l. in Shaanxi). The species occurs in various types of forests (evergreen broadleaved, deciduous broadleaved, mixed coniferous and evergreen broadleaved, and coniferous forests) as well as in orchards, where it was collected mainly from sifted samples of moist decaying debris of vegetation (leaf litter, humus, rotting wood) with fungi, and occasionally even from chicken excrement (Cuccodoro 2011).

### Megarthrus hemipterus

Taxon classificationAnimaliaColeopteraStaphylinidae

(Illiger, 1794)

602F4690-1850-57AF-B7C0-3D6DE2D14DA1

**Figures 7–9.**
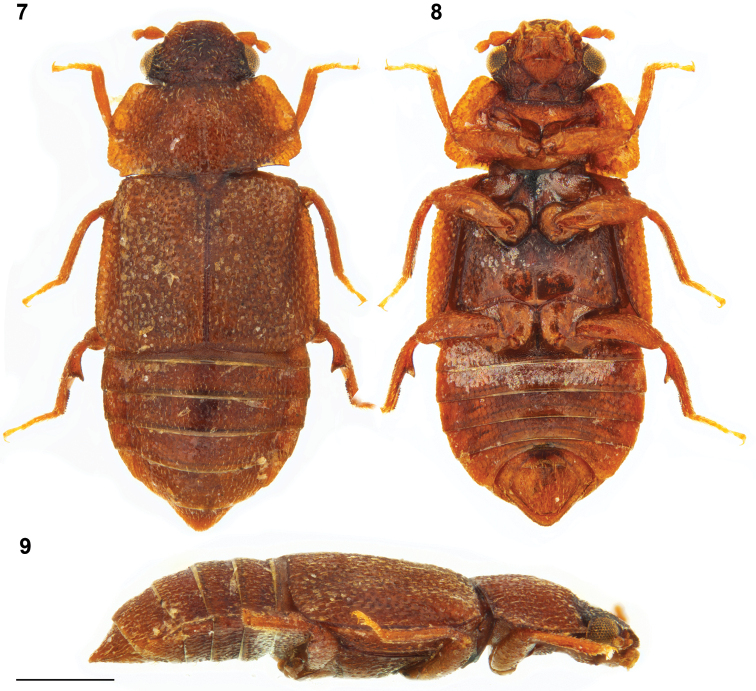
*Megarthrushemipterus* (Illiger): habitus, male: dorsal **7** ventral **8** and lateral **9** views. Scale bar: 0.5 mm.

Figs 7–9 , 12 , 15 , 18 , 41

Silpha hemiptera Illiger, 1794: 597; Cuccodoro 1996: 485 (new synonymy, Japanese records); Cuccodoro and Löbl 1997 (synonymic framework, detailed redescription, general distribution, the first record for China); Cuccodoro et al. 2011 (synonymic framework, photograph of dorsal habitus, records in Korea).

#### Diagnosis.

For detailed morphology see Cuccodoro and Löbl (1997). Combined length of head, pronotum, and elytra 1.6–1.9 mm; maximal pronotal width = 0.9–1.1 mm. Body and appendages (Figs 7–9) rust brown; head markedly darker with frons slightly paler than vertex. Anterior frontal margin slightly carinate, evenly convex in dorsal view.

***Male*.** Protibia fairly straight and slightly enlarged toward apex, evenly, with adventral side markedly depressed transversely at middle. Mesotrochanter with about a dozen of peg-like setae arranged in two rows. Mesofemur slightly arcuate and slightly swollen. Mesotibia subangulate, bearing peg-like setae arranged in one row. Metatrochanter and metafemur slightly swollen; posterior margin of metatrochanter evenly arcuate; inner margin of metafemur slightly concave in ventral view, forming sharp ridge on entire length. Metatibia swollen, at middle forming conspicuous tooth-like process projecting above flattened apical portion of metatibia; metatibial peg-like setae arranged in 1–2 rows on apical third, the latter group of 15–20, discontinuously with more than 12 peg-like setae arranged in two rows on distal side of tooth-like process; proximal side of tooth-like process broad and flat, bearing 4–10 scattered peg-like setae. Aedeagus (Figs 12, 15) with ventral wall not narrowed at base and gradually narrowed to mucronate apex in ventral view, with ventral fairly straight to apex in lateral view.

***Female*.** Gonocoxal plate with lateral portions of dorsobasal margin straight to middle portion forming small blunt process slightly projecting anterad. Dorsal part of genitalia with arcuate sclerite wider at middle (Fig. 18).

**Figures 10–18.**
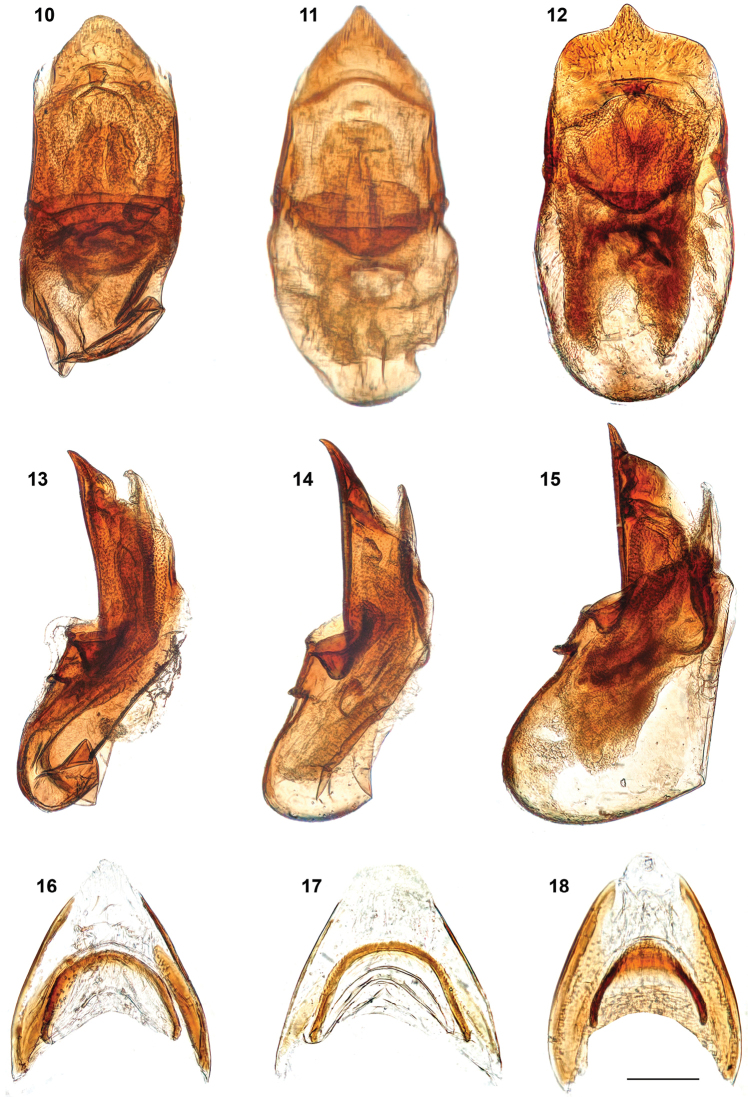
*Megarthrusdentipes* Bernhauer (**10, 13, 16**) *M.flavolimbatus* Cameron (**11, 14, 17**) *M.hemipterus* (Illiger) (**12, 15, 18**) male: aedeagus in ventral (**10–12**) and lateral (**13–15**) views female: genital segments, dorsal part (**16–18**) in ventral view. Scale bars: 0.1 mm.

#### Comparisons and diagnostic notes.

See above under *M.dentipes*.

#### Material examined.

(4 specimens): **China: Beijing**: ca 1400 m, Dongling Mts, Xiaolongmen, Liu Lang Yu, 39.97N, 115.43E, 15.vi.2001, leg. J. Cooter & P. Hlavá, mixed woodland litter, 1 ♀ in MHNG; **Heilongjiang Prov.**: “Süd-Mandshurei Chi-Kuan Shan, leg K. Rost”, 2 ♂ and 1 ♀ in FMNH.

#### Distribution and natural history.

*Megarthrushemipterus* is the most widespread member of the genus, with records ranging from the United Kingdom in the West to China, South Korea, Japan, and Far East Russia in the East (Cuccodoro 1996; Cuccodoro and Löbl 1997; Cuccodoro et al. 2011). It occurs in a wide range of microhabitats (carrion, fungi, leaf litter, and various types of decaying organic matter); in South Korea it was abundantly collected from fresh and rotten fungi. The first record for China without precise locality data (Cuccodoro and Löbl 1997) refers to the three specimens cited here from Chi-Kuan Shan from the Max Bernhauer’s collection, housed in the FMNH. The species, which occurs on most land masses surrounding the Sea of Japan, seems to reach its southern limit of extension in China in the Western Hills (Fig. 41), where it was collected in mid-April at 1400 m a. s. l. from mixed woodland litter on Mount Dongling (Beijing).

### Megarthrus panda sp. nov.

Taxon classificationAnimaliaColeopteraStaphylinidae

C8EE3A09-F60A-5DA7-BB1C-935EF88852DA

http://zoobank.org/536B7DD8-8691-4D7F-84AC-3BA210384AE1

Figs 19–21 , 22–23 , 24–32 , 33–40

#### Type material.

***Holotype*** (♂): **China: Yunnan Prov.**: Jizu Shan 25.58N 100.21E, 2500–2700 m, 6–10.vii.1994, leg. V. Kubáñ, in NHMB. ***Paratypes*** (20): Same data as holotype, 1 ♂ and 1 ♀ in NHMB, 1 ♂ and 1 ♀ in MHNG; **China: Yunnan Prov.**: NE Kunming, 25°08'35"N, 102°53'49"E, 2320 m, 13.viii.2014, leg. M. Schülke [CH14–06], mixed forest with alder, oak and pine, litter and mushrooms, sifted, 2 ♂ and 3 ♀ in cSch, 1 ♂ and 1 ♀ in MHNG & 1 ♂ and 1 ♀ in SWUC; NE Kunming, 25°08’40" N, 102°53’48"E, 2290 m, 11.viii.2014, leg. M. Schülke [CH14–05], mixed deciduous forest with scattered pine trees, litter and mushrooms sifted, 1 ♂ in cSch; Dali Aut. Pref. Mão Jiao Shan, E pass, 58 km NE Dali, 25°56’41"N, 100°40’05"E, 2525 m, 4.ix.2009, leg. M. Schülke [CH 09–26] secondary mixed forest, litter, moss & mushrooms, sifted, 1 ♂ and 4 ♀ in cSch & 1 ♀ in MHNG.

#### Description.

**Figures 19–21.**
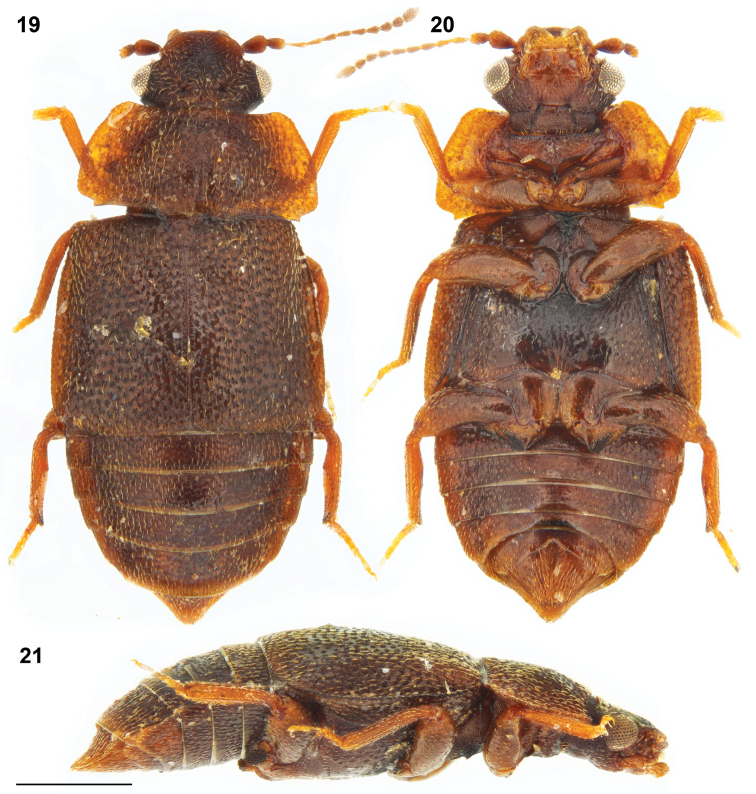
*Megarthruspanda* sp. nov.: habitus, male: dorsal **19** ventral **20**, and lateral **21** views. Scale bar: 0.5 mm.

**Figures 22–23.**
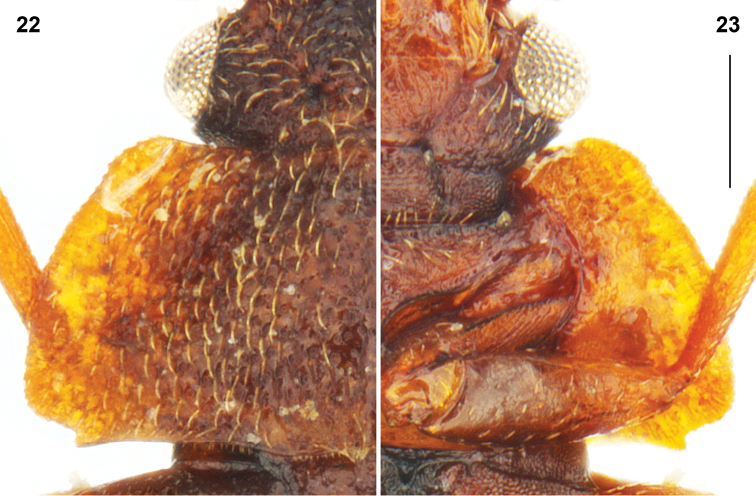
*Megarthruspanda* sp. nov., prothorax in dorsal **22** and ventral **23** views. Scale bar: 0.2 mm.

Combined length of head, pronotum and elytra = 1.7–2.1 mm; maximal pronotal width = 0.9–1.3 mm. Body (Figs 19–21) predominantly chestnut brown, with pronotum usually slightly paler, frons slightly paler than vertex, and legs slightly paler than elytra. Anterior frontal margin slightly carinate, evenly convex in dorsal view. Antenna as in Fig. 36. Prothorax as in Figs 22, 23.

**Figures 24–32.**
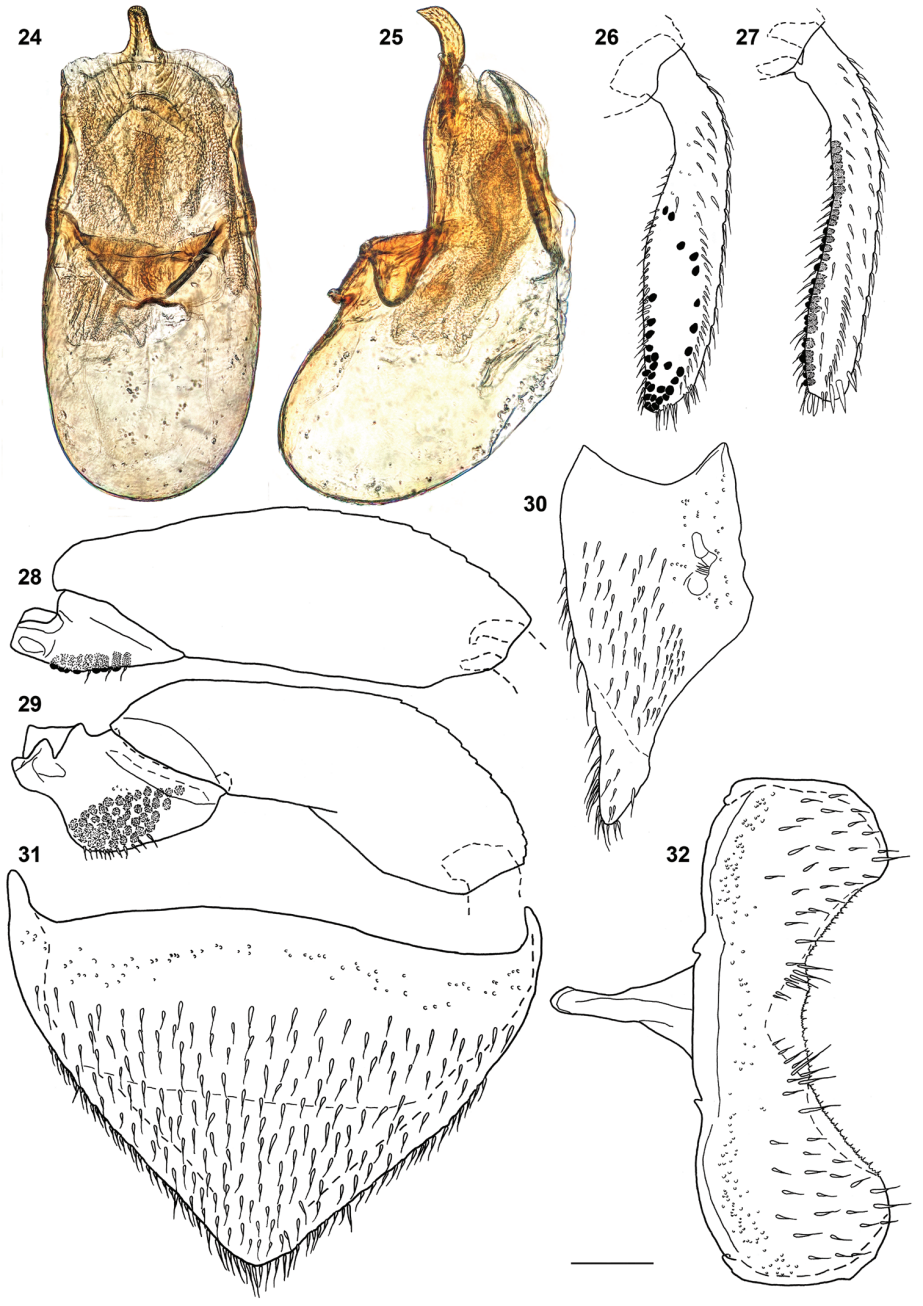
*Megarthruspanda* sp. nov., aedeagus in ventral **24** and lateral **25** views; metatibiae **26** mesotibiae **27** mesotrochanter and mesofemur **28** metatrochanter and metafemur **29** tergite VIII in lateral **30** and ventral **31** views sternite VIII in ventral view **32**. Scale bar: 0.1 mm.

***Male*.** Protibia fairly straight and evenly expanding from base to apex; adventral side flattened. Mesotrochanter (Fig. 28) with about twenty peg-like setae grouped as a field. Mesofemur (Fig. 28) slightly arcuate and slightly swollen. Mesotibia (Fig. 27) subangulate, bearing peg-like setae arranged in two rows. Metatrochanter and metafemur (Fig. 29) markedly swollen; posterior margin of metatrochanter broadly rectangular; posterior margin of metafemur concave in ventral view, forming sharp ridge on half length. Metatibia (Fig. 26) swollen, with adventral side flattened and broadly emarginated on apical two-thirds; metatibial peg-like setae grouped as a field on apical quarter with additional peg-like setae arranged in scattered row bordering each side of emargination. Abdominal tergite VIII as in Figs 30, 31; abdominal sternite VIII in Fig. 32; hemitergites IX as in Fig. 39. Aedeagus (Figs 24, 25) with ventral wall strongly narrowed at apical third in ventral view, with ventral outline markedly sinuate to slender apex strongly recurved ventrally in lateral view.

**Figures 33–40.**
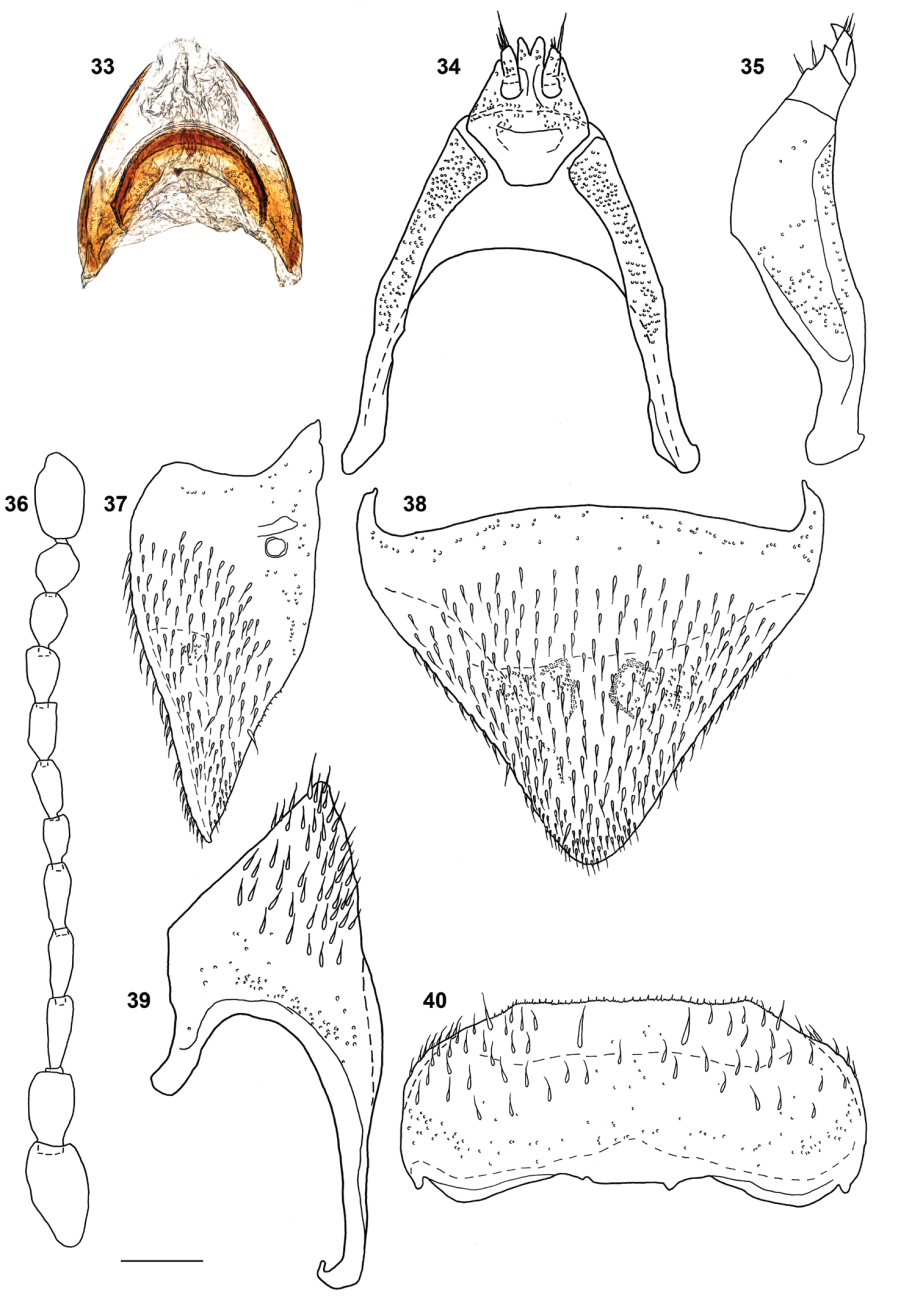
*Megarthruspanda* sp. nov., female genital segments, dorsal part in ventral view **33** and ventral part in dorsal **34** and lateral **35** views antenna **36** female tergite VIII in lateral **37** and dorsal **38** views male left hemitergite IX **39** female sternite VIII in ventral view **40**. Scale bar: 0.1 mm.

***Female*.** Abdominal tergite VIII as in Figs 37, 38. Valvifers as in Figs 34, 35. Gonocoxal plate (Figs 34, 35) with lateral portions of dorsobasal margin oblique to median portion truncate, markedly projecting anterad. Dorsal part of genitalia (Fig. 33) with arcuate sclerite slightly wider at middle.

#### Comparisons and diagnostic notes.

*Megarthrusdentipes*, *M.flavolimbatus*, *M.hemipterus* and *M.panda* sp. nov. are the only members of the genus to have the anterior frontal margin carinate, the antennae bearing short and dense pubescence only on antennomeres 5–11, hemispherical eyes with the highest point above level of the vertex, the lateral sides of pronotum and elytra slightly deplanate, the prohypomera without a marked ridge, and the pubescence on abdominal tergites parallel. Within these species *M.panda* sp. nov. can be easily distinguished by the shape posterior legs of the males (Figs 26, 29). Its genitalia are also diagnostic, notably in the male by the aedeagal ventral wall strongly sinuate in lateral view (Fig. 25), and in the female by the arcuate dorsal genital sclerite slightly wider at middle in combination with the dorsobasal margin of the gonocoxal plate evenly narrowed to its truncate median portion projecting anterad (Fig. 34).

#### Distribution and natural history.

**Figure 41.**
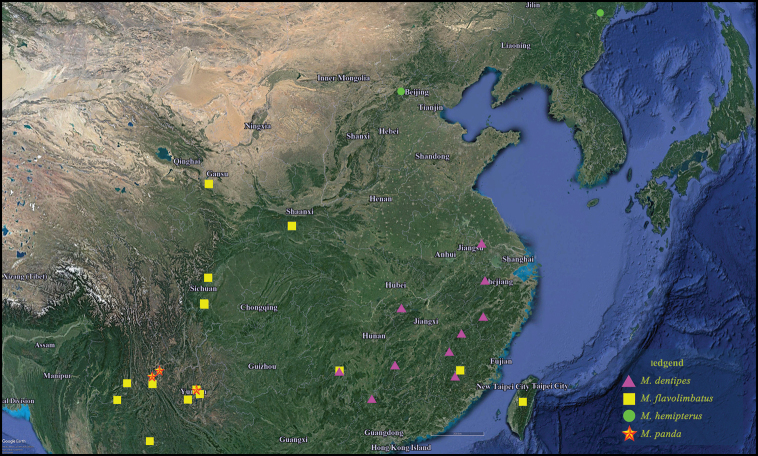
Distribution map in China of *Megarthrusdentipes* Bernhauer, *M.flavolimbatus* Cameron, *M.hemipterus* (Illiger) and *Megarthruspanda* sp. nov. Scale bar: 300 km.

The species is endemic to Yunnan Province (Fig. 41), where it was found at elevations ranging from 2290 to 2700 m a. s. l. from July to September by sifting leaf litter with mushrooms in mixed deciduous forests with alder, oak and pine.

#### Etymology.

*Megarthruspanda* sp. nov. shares the main body color and the forested mountains of Yunnan Province with its eponymous mammal the small panda, *Ailurusfulgens* Cuvier, 1825. Noun in apposition.

## Discussion

Cuccodoro (2011) grouped *M.dentipes*, *M.flavolimbatus* and *M.hemipterus* in the informal *M.hemipterus* complex defined as a subset of species of the *M.depressus* supergroup sharing strongly resembling male genitalia and metatibiae, and the female abdominal tergite VIII not forming a medioapical projection. In light of this new species, we refine the delimitation of this lineage as the members of the *M.depressus* supergroup (see Cuccodoro 2011: 89) possessing: 1) hemispherical eyes with highest point above level of vertex, 2) the dorsal valve of the aedeagus elongate, extended anteriorly beyond the apex of parameres, 3) the base of parameres projecting posteriorly above ventral wall, forming a cavity, and 4) the apex of the female abdominal tergite VIII acutely angled in lateral view, not forming a medioapical projection.

Among the four members of the *M.hemipterus* complex, i.e., *M.dentipes*, *M.flavolimbatus*, *M.hemipterus* and *M.panda* sp. nov., the male sexual dimorphism shows trends towards 1) moderate enlargement of the protibia with presence of a transverse adventral depression, 2) conspicuous enlargement of the metafemur and metatrochanter, and 3) presence of conspicuous tooth-like metatibial process. However, the precise phylogenetic relationships between these species have not been investigated.

The *M.hemipterus* complex includes both the most widespread *Megarthrus* species in the Palaearctic (*M.hemipterus*) and Oriental (*M.flavolimbatus*) realms, with its four constitutive members occurring in China. This country is also the only to host all species of this lineage, suggesting this area might have been its center of origin.

## Supplementary Material

XML Treatment for Megarthrus dentipes

XML Treatment for Megarthrus flavolimbatus

XML Treatment for Megarthrus hemipterus

XML Treatment for Megarthrus panda
